# MicroRNA-29b-3p Promotes Human Retinal Microvascular Endothelial Cell Apoptosis via Blocking SIRT1 in Diabetic Retinopathy

**DOI:** 10.3389/fphys.2019.01621

**Published:** 2020-01-29

**Authors:** Yong Zeng, Zekai Cui, Jian Liu, Jiansu Chen, Shibo Tang

**Affiliations:** ^1^Aier School of Ophthalmology, Central South University, Changsha, China; ^2^Aier Eye Institute, Changsha, China; ^3^Key Laboratory for Regenerative Medicine, Ministry of Education, Jinan University, Guangzhou, China; ^4^Institute of Ophthalmology, Medical College, Jinan University, Guangzhou, China; ^5^Center for Excellence in Brain Science and Intelligence Technology, Chinese Academy of Sciences, Beijing, China

**Keywords:** diabetic retinopathy, miR-29b-3p, SIRT1, human retinal microvascular endothelial cell, apoptosis

## Abstract

**Background:**

Diabetic retinopathy (DR) is a main complication of diabetes mellitus (DM). Recent studies have implicated microRNAs in human retinal microvascular endothelial cell (HRMEC) dysfunction. In this study, we aim to investigate the apoptotic promotion of miR-29b-3p by blocking SIRT1 in HRMEC for DR situation.

**Method:**

Blood samples were obtained from DR patients and controls. Dual-luciferase reporter assay using HEK-293T cells was performed to show the direct interaction of miR-29b-3p and the 3′UTR of SIRT1. HRMECs were exposed to 5.5 mmol/L of glucose (normal control), 5.5 mmol/L of glucose and 24.5 mmol/L of mannitol (osmotic pressure control), 30 mmol/L of glucose [hyperglycemia (HG)], 150 μmol/L of CoCl_2_ (hypoxia), and 30 mmol/L of glucose plus 150 μmol/L of CoCl_2_ (HG-CoCl_2_). To identify the regulating relationship between miR-29b-3p and SIRT1, HRMECs were transfected with miR-29b-3p mimics/inhibitors or their negative controls. SRT1720 was used as a SIRT1 agonist. Cell viability was assessed with the cell counting kit-8 (CCK-8) assay, and apoptotic cells were stained by one-step terminal deoxynucleotidyl transferase dUTP nick end labeling (TUNEL) assay kit. Gene and protein expression were assayed by quantitative real-time reverse transcriptase-PCR (RT-qPCR) and western blotting separately.

**Result:**

MiR-29b-3p was upregulated to 3.2-fold, and SIRT1 protein was downregulated to 65% in DR patients. Dual-luciferase reporter assay showed the direct interaction of miR-29b-3p and SIRT1. HRMECs were identified as >95% positive for CD31 and von Willebrand factor (vWF). MiR-29b-3p and Bax/Bcl-2 ratio was upregulated, whereas SIRT1 was downregulated in HRMECs in the HG-CoCl_2_ condition. Decreased cell viability and upregulated apoptosis were also found in HRMECs of the HG-CoCl_2_ condition. Upregulated miR-29b-3p decreased the expression of SIRT1 and increased the ratio of Bax/Bcl-2, whereas downregulated miR-29b-3p increased the expression of SIRT1 protein and downregulated the ratio of Bax/Bcl-2. SRT1720 rescued miR-29b-3p-induced HRMEC apoptosis via upregulating the expression of SIRT1 protein.

**Conclusion:**

The dysregulation of miR-29b-3p/SIRT1 is a potential mechanism of HRMEC apoptosis in DR. MiR-29b-3p/SIRT1 may be a potential therapeutic target for DR.

## Introduction

Diabetic retinopathy (DR) is a main complication of diabetes mellitus (DM), and it is a leading cause of blindness in working-aged adults worldwide (Chong et al., 2017). Vascular damage, which may be caused by cell apoptosis, inflammation, oxidative stress, and a series of metabolic disorders, is an initial characteristic and will exacerbate DR progression (Kannenkeril et al., 2018; Miloudi et al., 2019). Once the blood–retina barrier is broken down, dangerous factors from the circulating blood will leak into the retinal tissue and cause irreversible damage to the retinal neural cells (Trost et al., 2016; Xu and Chen, 2017). Although a series of studies have been carried out to investigate the pathogenesis of human retinal vascular endothelial cell apoptosis (Santiago et al., 2018; Whitehead et al., 2018), the mechanism is largely unknown.

MicroRNA is a kind of non-coding RNA composed of 19–25 nucleotides (Lu and Rothenberg, 2018). Thousands of microRNAs have been discovered since they were first reported in 1993, and a single kind of microRNA may have hundreds of target mRNAs (O’Kelly et al., 2012; Landrier et al., 2019). MicroRNA regulates post-transcriptional gene expression via binding to target sites directly or promoting mRNA degradation (Landrier et al., 2019). A series of studies have revealed the regulation of microRNA in aging, tumor progression, metabolic diseases, and inflammation (Klieser et al., 2019; Majidinia et al., 2019; Nasr et al., 2019; Zhao et al., 2019). In recent years, a variety of microRNAs (miR-409-3p, miR-98-5p, miR-16-5p, etc.) have been proved to participate in DM progression and its complications (Duan et al., 2019; Khan et al., 2019; Ventriglia et al., 2019). Furthermore, numerous microRNAs (miR-3197, miR-2116-5p, miR-152, miR-34a, etc.) are identified as specificity biomarkers, and they participate directly in DR progression (Fu and Ou, 2019; Ji et al., 2019; Thounaojam et al., 2019). SIRT1 is a NAD^+^-dependent protein deacetylase, which plays important roles in metabolic regulation and adaptation (Boutant and Cantó, 2014). Through deacetylation of various transcription factors (p53, p65, STAT3, etc.), SIRT1 widely takes part in the regulation of inflammation, oxidative stress, autophagy, and cell apoptosis (Kitada et al., 2019; Sanz et al., 2019). Researchers believe that SIRT1 is a protective factor in DM and its complications (Potente et al., 2007; Boutant and Cantó, 2014; Collin et al., 2019; Myers et al., 2019). Bax is widely accepted as a pro-apoptosis factor, whereas Bcl-2 is an anti-apoptosis factor; the ratio of Bax/Bcl-2 may be more important than either alone in determining apoptosis (Oltvai et al., 1993). SIRT1 is proved to downregulate the ratio of Bax/Bcl-2, thus further attenuating cell apoptosis (Nguyen et al., 2019; Sasaki et al., 2019).

Recent studies have revealed the decrease of SIRT1 in diabetes patients (Balestrieri et al., 2013) and the increase of miR-29b-3p in diabetes (Esteves et al., 2018). Furthermore, Su et al. (2019) have proved the direct regulation of miR-29b-3p to SIRT1 in insulin resistance. To our knowledge, there is no research to illuminate the regulatory relationship of miR-29b-3p/SIRT1/Bax/Bcl-2 pathway in human retinal microvascular endothelial cell (HRMEC). Here, we performed this study to verify the role of miR-29b-3p in DR.

## Materials and Methods

### Patients and Tissues

This study was conducted in accordance with the Declaration of Helsinki and the guidelines of the Ethics Committee of Aier Eye Hospital (Changsha, Hunan, China). This study was approved by the ethics committee of Aier Eye Hospital (AIER2018IRB21) and registered on the International Clinical Trials Registry Platform (ChiCTR1900025449). Consent was obtained from all the participants before collection. Blood samples were obtained from 21 DR patients aged 37–71 years. Negative control (NC) blood samples came from 11 pterygium patients without DM aged 53–69 years. All the samples were stored at −80°C for further experiments. Human retinas in this study were obtained from organ donors without DM or retinal diseases.

### Dual-Luciferase Reporter Assay

We predicted SIRT1 as a potential target of miR-29b-3p by using miRNA database (TargetScanHuman 7.2). Then the 3′-UTR of human SIRT1 containing the predicted binding sites [wild type (WT)] or mutated binding sites [mutant type (MUT)] was amplified and inserted into pmir-RB-Report^TM^ vector. The reporter plasmids and miR-29b-3p mimics or NC were co-transfected into HEK-293T cells using Lipofectamine 2000 (Invitrogen) to determine if SIRT1 is a direct target of miR-29b-3p. Firefly and Renilla luciferase activities were measured 48 h after transfection using the Dual-Glo^®^ Luciferase Assay System (Promega, Madison, WI, United States).

### Cell Culture and Treatment

Primary HRMECs were isolated according to methods described previously (Fan et al., 2016); briefly, retinas were immersed in phosphate-buffered saline (PBS) with 5% penicillin–streptomycin (Gibco, Thermo Fisher Scientific, Waltham, MA, United States) for 5 min and then transferred into Dulbecco’s modified Eagle’s medium (DMEM) to remove vitreous. Retinas were then minced into small pieces and digested with type II collagenase (Sigma Aldrich, St. Louis, MO, United States) at 37°C for 30 min. Cell suspension was filtered through a 70-μm mesh and cultured in endothelial cell medium (ECM; ScienCell, San Diego, CA, United States). Culture medium was replaced every 2–3 days.

To explore the HRMEC damage in DR, we constructed an *in vitro* model of hyperglycemia and hypoxia conditions. HRMECs were cultured in 5.5 mmol/L of glucose (normal control), 5.5 mmol/L of glucose and 24.5 mmol/L of mannitol (osmotic pressure control), 30 mmol/L of glucose [hyperglycemia (HG)], 150 μmol/L of CoCl_2_ (hypoxia), 30 mmol/L of glucose, and 150 μmol/L of CoCl_2_ (HG-CoCl_2_). Culture medium was refreshed every 24 h. SRT 1720 Hydrochloride (MedChemExpress, Monmouth Junction, NJ, United States) was used as an activator to upregulate the expression of SIRT1.

### Immunofluorescence

Immunofluorescence to platelet endothelial cell adhesion molecule-1 (PECAM-1/CD31) and von Willebrand factor (vWF) were used to determine the endothelial cell purity (Gao et al., 2013). Primary antibodies to CD31 (mouse anti-CD31 antibody, ab24590, 1:100, Abcam) and vWF (rabbit polyclonal to vWF antibody, ab6994, 1:100, Abcam) were used to detect CD31 and vWF, respectively. Goat anti-mouse IgG secondary antibody (Alexa Fluor 594) and goat anti-rabbit IgG secondary antibody (Alexa Fluor 488) were used to detect the primary antibodies separately. Nuclei were stained with DAPI (blue). Cells of passages between 3 and 5 and 95% positive for CD31 and vWF were used in this study.

### Cell Transfection

Cells were seeded in 6-well and 96-well plates with a density of 2 × 10^5^/well and 4 × 10^3^/well. The miR-29b-3p mimics, inhibitors, and their NCs were purchased from RiboBio (Guangzhou, China) and transfected into cells using riboFECT^TM^ CP Reagent (Guangzhou, China) according to the manufacturer’s protocols. NC mimics labeled with Cy3 fluorescence (Guangzhou, China) were transfected to observe the transfect efficiency directly. After 30 h of transfection, the HRMECs were collected for terminal deoxynucleotidyl transferase dUTP nick end labeling (TUNEL) stain, cell counting kit-8 (CCK-8), quantitative real-time reverse transcriptase-PCR (RT-qPCR), and Western blot (WB) assay.

### Cell Apoptotic and Viability Assay

For apoptosis and viability assay, 4 × 10^3^ cells/well were seeded into 96-well plates and cultured at 37°C with 5% CO_2_ in a humidified environment. The One Step TUNEL Apoptosis Assay Kit (Beyotime) was used for detecting apoptotic cells. Nuclei were stained with DAPI (blue). Fluorescent images were acquired by a fluorescence microscope (ECLIPSE Ts2R, Nikon). The quantification of TUNEL-positive cells was obtained by ImageJ software and calculated by GraphPad Prism version 5.0. Cell viability was determined by a CCK-8 assay (MedChemExpress, Monmouth Junction, NJ, United States). Seven replicates per group and a group without cells served as the blank. After being treated with different conditions, 100 μl of fresh culture medium with 10% CCK-8 solution was added to each well and incubated at 37°C for 1.5 h. The absorbance at 450 nm was observed by Synergy^TM^ HTX Multi-Mode Microplate Reader (Bio-Tek Technologies, Winooski, VT, United States). The relative viability of cells was calculated according to the manufacturer’s protocol.

### Quantitative Real-Time Reverse Transcriptase–Polymerase Chain Reactions

MicroRNA was isolated with a microRNA kit (Omega Bio-Tek, Norcross, GA, United States) and reversed to cDNA with a reverse transcription kit (Roche, Basel, Switzerland); the stem-loop method was especially used for microRNA reverse transcription as described previously (Chen et al., 2005). The RT product was subjected to 45 cycles of qPCR reactions with ChamQ Universal SYBR qPCR Master Mix (Vazyme Biotech, Jiangsu, China) in a Roche LightCycler^®^ 96 System (Roche, Basel, Switzerland). U6 was used to normalize the expression of microRNA. The relative expression level of miRNA was calculated by the 2^–Δ^ ^Δ^ ^CT^ method. The specific primers for miR-29b-3p and U6 are listed in Table 1.

**TABLE 1:** The sequences of specific primers.

**Human genes**		**Primer sequences**
MiR-29b-3p	Stem-loop primer	GTCGTATCCAGTGCAGGG TCCGAGGTATTCGCAC TGGATACGACAACACTGA
	Forward primer	CTGCTAGCACCATTTGAAA
	Reverse primer	GTGCAGGGTCCGAGGT
U6	Forward primer	CTCGCTTCGGCAGCACA
	Reverse primer	AACGCTTCACGAATTTGCGT
		

### Western Blots

After being treated with different conditions, cells were washed twice with ice-cold PBS and lysed with radioimmunoprecipitation assay (RIPA) buffer (Beyotime) supplemented with protease inhibitor cocktail (Sigma-Aldrich). Lysates were then centrifuged at 12,000 rpm for 20 min at 4°C to collect the supernatant. Protein quantification was performed using BCA Protein Assay Kit (Solarbio) according to the company’s protocol. The supernatant proteins were concentrated with the method described previously (Zaiss et al., 2013). Briefly, supernatant, methanol, and chloroform were mixed thoroughly. The mixture was centrifuged at 10,000 rpm for 10 min at 4°C, and the supernatant was discarded carefully. Then another volume of methanol was added to the pellet and vortexed to mix thoroughly. The mixture was again centrifuged at 12,000 rpm for 10 min at 4°C, and the supernatant was discarded. After being air-dried for 5 min, the proteins were dissolved with the lysis buffer from a Caspase 3 Activity Assay Kit (Beyotime), and the quantification was performed using a Bradford Protein Assay Kit (Solarbio).

Protein was denatured using sodium dodecyl sulfate–polyacrylamide gel electrophoresis (SDS-PAGE) loading buffer (Solarbio) by heating the samples at 98°C for 6 min. Electrophoresis was performed using 10% SDS-PAGE gel and transferred onto nitrocellulose membranes (Pall) and blocked with 5% non-fat milk containing Tween-20 for 1 h at room temperature, followed by incubation with primary antibodies overnight at 4°C. IRDye^®^ 800CW goat anti-rabbit/mouse secondary antibody (LI-COR) was used to detect primary antibody binding. The immunoblots were analyzed and quantified using ImageJ software. Antibodies to SIRT1(19A7AB4), Bax (E63), and Bcl-2 (E17) were obtained from Abcam. Caspase-3 and β-actin (8H10D10) antibodies were obtained from Cell Signaling Technology. Total protein stain was performed by using a REVERT Total Protein Stain kit (LI-COR). Relative quantification of cleaved caspase-3 in supernatant was achieved by normalizing each target to the value of total proteins.

### Statistical Analysis

Statistical Package for Social Science (SPSS) software version 19.0 and GraphPad Prism version 5.0 were used for descriptive analysis. The data were shown as mean ± standard deviation (SD). The results presented in the paper were representative of at least three different repetitions. Student’s *t* test was performed to assess differences between two means. A chi-square test for qualitative data was applied. One-way or two-way ANOVA followed by Bonferroni’s *post hoc* test was performed in multiple means comparison. Statistical significance was defined as *p* < 0.05.

## Results

### SIRT1 Is a Direct Target of MiR-29b-3p in Diabetic Retinopathy

**FIGURE 1:**
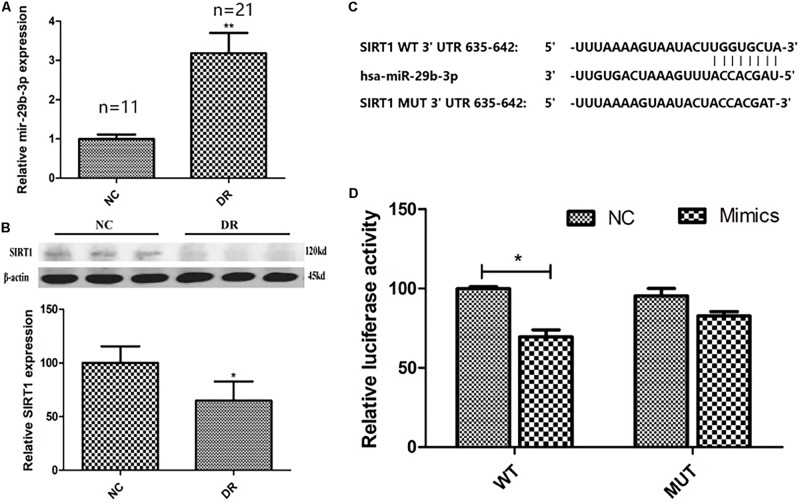
The clinical expression pattern and dual-luciferase reporter assay of miR-29b-3p and SIRT1. **(A)** In DR patients’ blood, miR-29b-3p was upregulated to 3.2-fold compared with that of control group. **(B)** DR patients’ blood SIRT1 protein was downregulated to 65% compared with that of control group. **(C)** SIRT1 is a direct target of miR-29b-3p predicted by the miRNA online database (TargetScanHuman7.2 and miRBase). **(D)** Dual-luciferase reporter assay using HEK-293T cells showed the direct interaction of miR-29b-3p and the 3′UTR of SIRT1. After 48-h cotransfection, overexpression of miR-29b-3p reduced the luciferase activity of WT reporter but had no inhibition on the MUT reporter. Data are shown as mean ± SD, *p < 0.05, **p < 0.01. NC, negative control; DR, diabetic retinopathy; WT, wild type; MUT, mutant type.

The baseline data of clinical samples are shown in Table 2. To explore the expression pattern of miR-29b-3p and SIRT1 in DR patients, RT-qPCR and WB were performed. MiR-29b-3p RNA was upregulated to 3.2-fold (Figure 1A), and SIRT1 protein was downregulated to 65% (Figure 1B) in DR patients’ blood samples. With the miRNA online database (TargetScanHuman7.2 and miRBase), we found that SIRT1 is a direct target of miR-29b-3p (Figure 1C). Dual-luciferase reporter assay using HEK-293T cells showed the direct interaction of miR-29b-3p and the 3′UTR of SIRT1. After 48-h cotransfection, overexpressed miR-29b-3p reduced the luciferase activity of WT reporter but had no inhibition on the MUT reporter (Figure 1D). The results of this study showed that miR-29b-3p could inhibit the expression of SIRT1 by binding with the 3′-UTR of SIRT1, and SIRT1 might be the downstream target gene of miR-29b-3p.

**TABLE 2:** Demographic and clinical characteristics of the study population.

**Variables**	**Controls**	**Patients**	***p***
Number	11	21	
**Age (years)**			
Mean ± SD	58.2 ± 5.5	56.8 ± 8.8	0.109
**Sex**			
Female	5 (45.5%)	13 (61.9%)	0.302
Male	6 (54.5%)	8 (38.1%)	
Weight (kg) Mean ± SD	60.0 ± 8.01	60.1 ± 10.4	0.337
Height (m) Mean ± SD	1.60 ± 0.05	1.62 ± 0.06	0.685
BMI	23.3 ± 2.4	22.8 ± 2.9	0.849
**Hypertension**			
Negative	9 (81.8%)	11 (52.4%)	0.104
Positive	2 (18.2%)	10 (47.6%)	
FPG (mmol/L)	4.55 ± 0.52	7.47 ± 2.49	0.001

*Data are represented as number (percentage) or mean ± standard deviation (SD). Patients are diabetic retinopathy patients. Student’s *t* test for quantitative variables and chi-square test for qualitative data were applied. BMI, body mass index; FPG, fasting plasma glucose.*

### Verification of Human Retinal Microvascular Endothelial Cell

**FIGURE 2:**
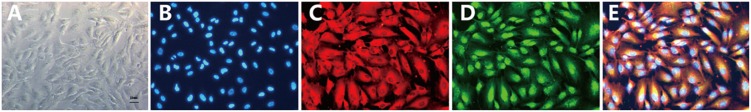
Culture and verification of HRMEC. **(A)** After 10-day culture, the cells showed an oval morphology and a contact-inhibited monolayer. **(B)** Cell nuclei were stained with DAPI (blue fluorescent). **(C)** CD31 was positive (red fluorescent). **(D)** vWF was positive (green fluorescent). **(E)** Merged picture revealed double staining positive for CD31/vWF. The cell morphology and immunofluorescent results verified the HRMEC cell type. HRMEC, human retinal microvascular endothelial cell; CD31, platelet endothelial cell adhesion molecule-1; vWF, von Willebrand factor.

Human retinal microvascular endothelial cell clusters began to form on the third day after plating. After 10-day culture, the cells showed an oval morphology and a contact-inhibited monolayer (Figures 2A,B). Immunofluorescence was performed to detect CD31 and vWF, which were well-known typical vascular endothelial cell markers. As a result, both CD31 (Figure 2C) and vWF (Figure 2D) were positive on the same cells (Figure 2E). These results verified the cell type and purification.

### Expression of MiR-29b-3p and SIRT1 in Human Retinal Microvascular Endothelial Cell Under Hyperglycemia–CoCl_2_ Condition

**FIGURE 3:**
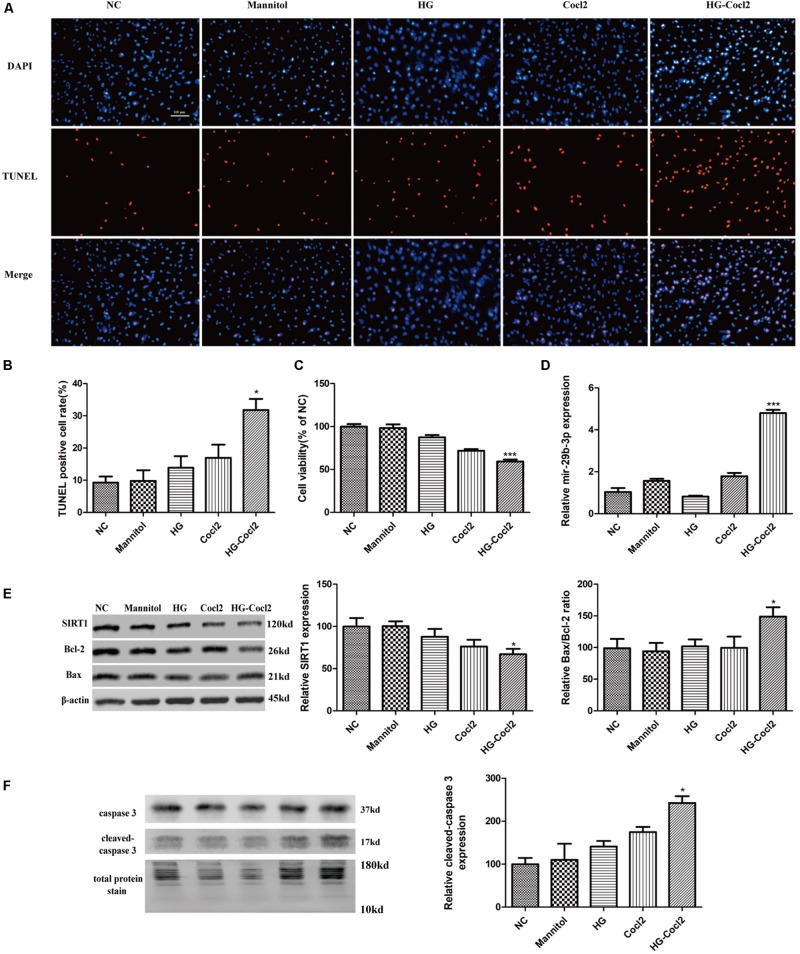
Cell apoptosis and expression pattern of miR-29b-3p/SIRT1 in HRMEC under HG-CoCl_2_ condition. **(A)** Obvious apoptotic cells were observed in HG-CoCl_2_ condition. **(B)** Quantification of TUNEL-positive cells. **(C)** Cell viability was decreased by HG-CoCl_2_ condition compared with that of different controls. **(D)** MiR-29b-3p was upregulated significantly in HG-CoCl_2_ condition. **(E)** SIRT1 protein was downregulated and Bax/Bcl-2 ratio was upregulated. **(F)** Relative expression of cleaved caspase-3 was upregulated by HG-CoCl_2_ treatment. Data are shown as mean ± SD, *p < 0.05 versus the NC group and ***p < 0.001 versus the NC group. NC, negative control; HG, high glucose; TUNEL, terminal deoxynucleotidyl transferase dUTP nick end labeling.

After treatment with the HG-CoCl_2_ condition and different controls, obvious apoptosis was observed by TUNEL assay (Figures 3A,B), and cell viability was decreased compared with that of controls (Figure 3C). MiR-29b-3p was upregulated (Figure 3D) in the HG-CoCl_2_ condition, whereas SIRT1 protein was downregulated (Figure 3E). HG-CoCl_2_ upregulated Bax/Bcl-2 ratio and the expression of cleaved caspase-3 significantly (Figures 3E,F).

### MiR-29b-3p Inhibits SIRT1 in Human Retinal Microvascular Endothelial Cell

**FIGURE 4:**
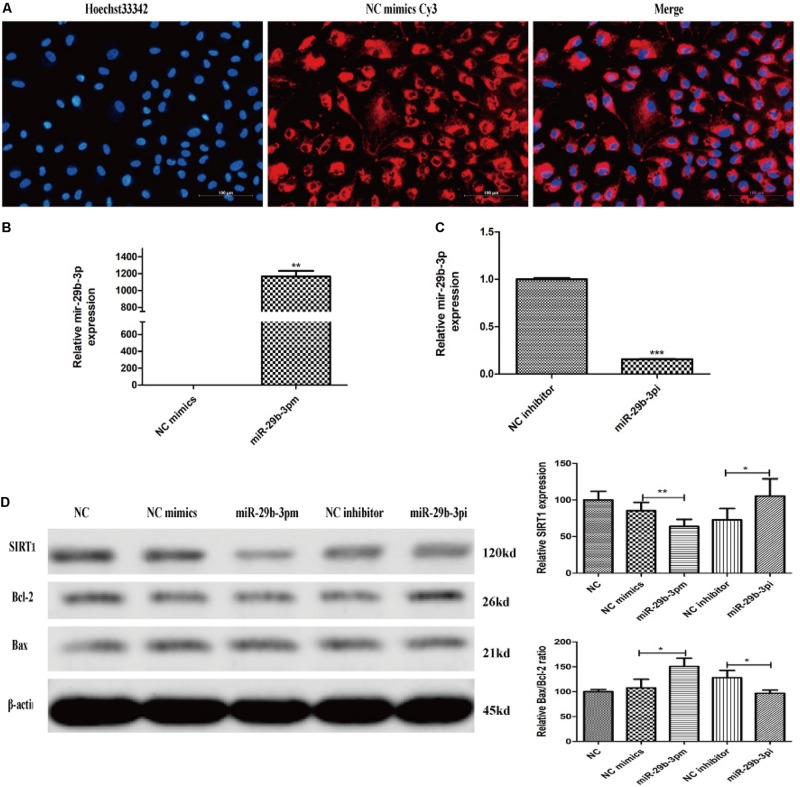
MiR-29b-3p mimics, inhibitors, and their negative controls were transfected into HRMEC successfully. **(A)** Annulus red fluorescence was observed around the nucleus after 30-h transfection with NC mimics Cy3. **(B)** The mRNA expression level of miR-29b-3p in miR-29b-3pm transfection was elevated significantly. **(C)** The mRNA expression level of miR-29b-3p was decreased in miR-29b-3pi transfection. **(D)** Relative SIRT1 protein expression was downregulated and the ratio of Bax/Bcl-2 was upregulated in miR-29b-3pm to negative control. In miR-29b-3pi, relative SIRT1 protein expression was upregulated and the ratio of Bax/Bcl-2 was downregulated versus that of negative control. Data are shown as mean ± SD. *p < 0.05, **p < 0.01, and ***p < 0.001. NC, negative control; miR-29b-3pm, miR-29b-3p mimics; miR-29b-3pi, miR-29b-3p inhibitor.

After 30-h transfection, annulus red fluorescence was observed around the nucleus (Figure 4A). HRMECs were transfected with miR-29b-3p mimics (miR-29b-3pm), inhibitors (miR-29b-3pi), and their NCs. RT-qPCR and WB were performed to verify the transfection effect. The mRNA expression level of miR-29b-3p in miR-29b-3pm was elevated (Figure 4B), whereas miR-29b-3pi decreased the expression of miR-29b-3p obviously (Figure 4C). Relative SIRT1 protein expression was downregulated and the ratio of Bax/Bcl-2 was upregulated in miR-29b-3pm to NC, whereas relative SIRT1 protein expression was upregulated and the ratio of Bax/Bcl-2 was downregulated in miR-29b-3pi to NC (Figure 4D).

### MiR-29b-3p Promotes Human Retinal Microvascular Endothelial Cell Apoptosis via Blocking SIRT1

**FIGURE 5:**
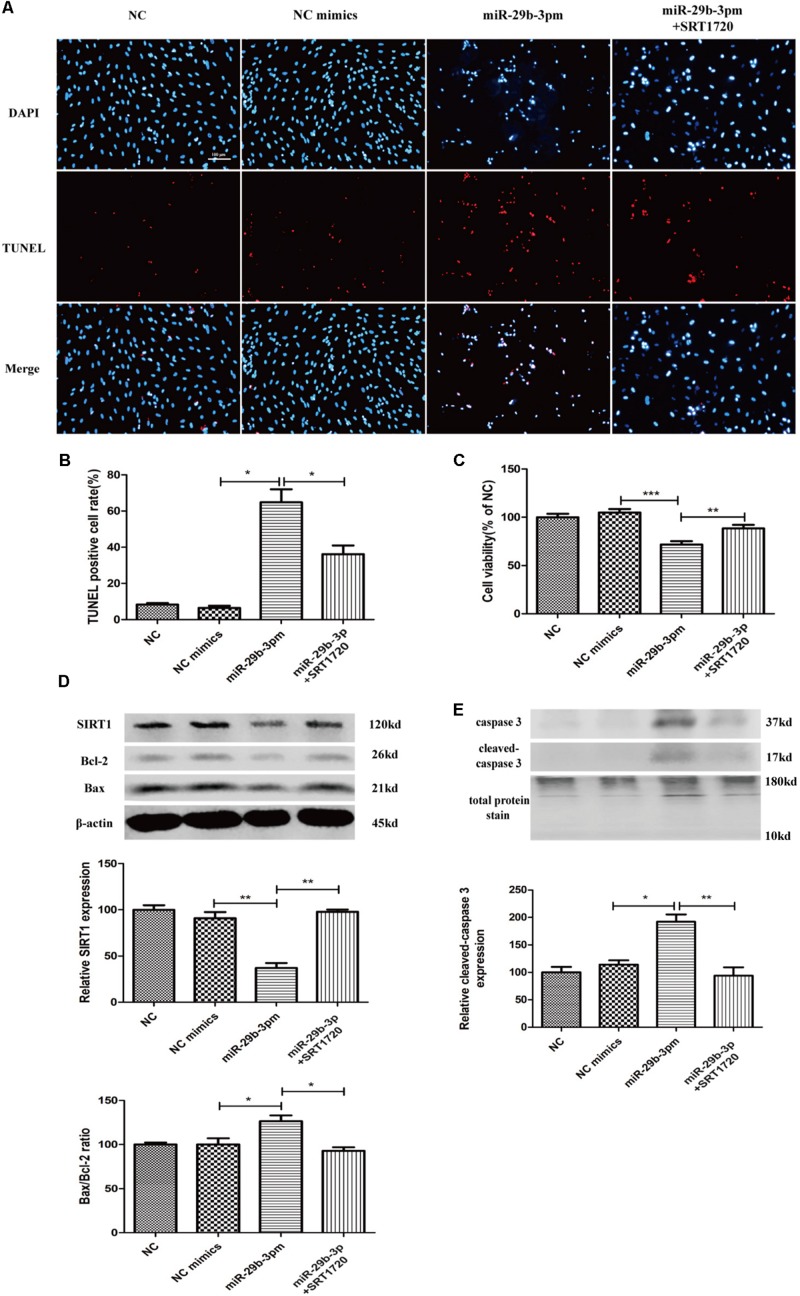
MiR-29b-3p promotes HRMEC apoptosis via blocking SIRT1. **(A)** Upregulated miR-29b-3p promoted apoptosis, whereas SRT1720 alleviated the apoptosis promotion of miR-29b-3p. **(B)** Quantification of TUNEL-positive cells. **(C)** Cell viability was downregulated by miR-29b-3pm and upregulated after the treatment of SRT1720. **(D)** Relative SIRT1 protein expression was decreased and Bax/Bcl-2 ratio was upregulated after the transfection of miR-29b-3pm, whereas after the treatment of SRT1720, relative SIRT1 protein expression was upregulated and Bax/Bcl-2 ratio was downregulated. **(E)** Relative cleaved caspase-3 protein expression was upregulated after the transfection of miR-29b-3pm and downregulated after the treatment of SRT1720. Data are shown as mean ± SD. *p < 0.05, **p < 0.01, and ***p < 0.001. NC, negative control; miR-29b-3pm, miR-29b-3p mimics; TUNEL, terminal deoxynucleotidyl transferase dUTP nick end labeling.

MiR-29b-3p mimics were transfected into HRMEC with or without SRT1720. Upregulated miR-29b-3p increased apoptosis (Figures 5A,B) and decreased cell viability in HRMEC (Figure 5C), whereas apoptosis was decreased (Figures 5A,B) and cell viability was upregulated after the treatment of SRT1720 (Figure 5C). Relative SIRT1 protein expression was decreased and Bax/Bcl-2 ratio and cleaved caspase-3 were upregulated after the transfection of miR-29b-3p, whereas after the treatment of SRT1720, relative SIRT1 protein expression was upregulated and Bax/Bcl-2 ratio and cleaved caspase-3 were downregulated (Figures 5D,E).

### MiR-29b-3pi and SRT1720 Alleviate Hyperglycemia–CoCl_2_-Induced Human Retinal Microvascular Endothelial Cell Apoptosis

**FIGURE 6:**
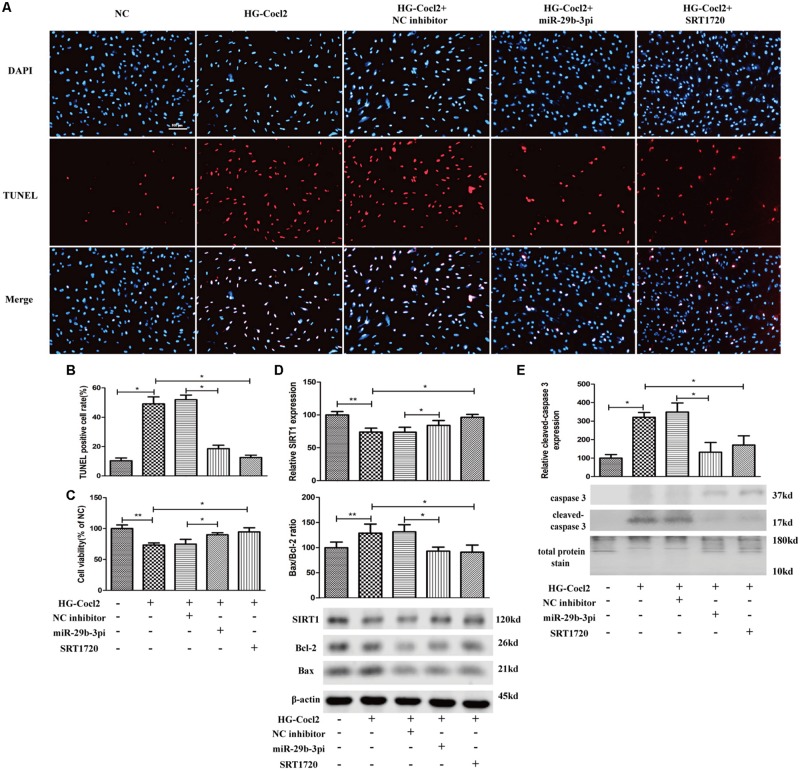
MiR-29b-3p inhibitor and SRT1720 alleviate HG-CoCl_2_-induced HRMEC apoptosis. **(A)** Cell apoptosis was induced by HG-CoCl_2_ obviously, whereas miR-29b-3pi and SRT1720 alleviated the apoptosis significantly. **(B)** Quantification of TUNEL-positive cells. **(C)** MiR-29b-3pi and SRT1720 promoted cell viability in HG-CoCl_2_ condition. **(D)** MiR-29b-3pi and SRT1720 increased the expression of SIRT1 protein and downregulated Bax/Bcl-2 ratio in HG-CoCl_2_ condition. **(E)** Relative expression of cleaved caspase-3 was downregulated by miR-29b-3pi and SRT1720. Data are shown as mean ± SD. *p < 0.05 and **p < 0.01. NC, negative control; HG-CoCl_2_, high glucose and CoCl_2_; miR-29b-3pi, miR-29b-3p inhibitor; TUNEL, terminal deoxynucleotidyl transferase dUTP nick end labeling.

MiR-29b-3pi and SRT1720 effectively alleviated HRMEC apoptosis induced by HG-CoCl_2_ (Figures 6A,B) and improved cell viability (Figure 6C). Relative SIRT1 protein expression was increased and Bax/Bcl-2 ratio (Figure 6D) and cleaved caspase-3 were also downregulated obviously by miR-29b-3pi and SRT1720 (Figure 6E).

## Discussion

Diabetic retinopathy is a serious complication of DM, which may cause irreversible vision loss or even blindness. Microvascular damage is a typical worsening characteristic as DM progresses (Stehouwer, 2018). However, the mechanism of microvascular damage in DR is complicated and should be further studied. In this study, we illuminated the dysregulation of miR-29b-3p/SIRT1 in DR patient blood samples. Additionally, using cultured HRMEC apoptotic model induced by HG-CoCl_2_, we proved that miR-29b-3p promoted HRMEC apoptosis. These findings indicate that miR-29b-3p may be an important regulator in vascular damage in DR progression.

Hyperglycemia and hypoxia are the leading causes of diabetic vascular injury via enhanced oxidative stress, nitrosative stress, and advanced glycation, thus promoting inflammation (Miyata and de Strihou, 2010; Chiu and Taylor, 2011). The experimental model of hyperglycemia and hypoxia conditions is widely used in the research of diabetic complications (Bakhashab et al., 2014, 2016). In this study, we constructed an *in vitro* hyperglycemia and hypoxia model with HRMEC to investigate the diabetic vascular damage mechanism. This model revealed similar apoptotic promotion as the study from Bakhashab et al. (2018) on human umbilical vein endothelial cells.

Previous studies revealed that dysregulation of miR-29 family promoted cell apoptosis in many human diseases including cerebral ischemia/reperfusion injury, pulmonary arterial hypertension, and myocarditis (Chen et al., 2018; Huang et al., 2018; Zhang et al., 2018). Yuan et al. (2018) found that miR-29b would activate NF-κB, thus aggravating endothelial cell inflammatory damage. Overexpression of miR-29b increased the expression of cleaved caspase-3, which is a typical apoptotic factor (Yuan et al., 2018). Saravanan et al. (2019) reported that miR-29b-3p was selectively released in exosomes from inflammation and hypoxia induced islets before apoptosis and cell death, which coincided with activation of endoplasmic reticulum (ER) stress response markers IRE-1α, XBP1, HIF-1α, and CHOP. They also detected the selective release of miR-29b-3p in plasma exosomes after these cells were transplanted into streptozotocin (STZ) diabetic nude mice. Therefore, miR-29b-3p might be an early indicator of human islet cell apoptosis during prediabetic conditions (Saravanan et al., 2019). MiR-29b-3p was obviously upregulated in high glucose-induced endothelial cells (Silambarasan et al., 2016), and overexpression of miR-29b-3p could cause insulin resistance in mice (Su et al., 2019). These findings indicate that miR-29b-3p may be involved in vascular damage and DM progression. Our findings in clinical samples and HG-CoCl_2_-induced HRMEC are consistent with these studies.

SIRT1 was proved to alleviate inflammation and apoptosis via deacetylating inflammatory transcription factors; therefore, SIRT1 was gradually coming to be interpreted as a DR protector (Mishra et al., 2018), whereas in DM patients and especially in patients with poor glycemic control, SIRT1 was significantly downregulated (Balestrieri et al., 2013). We also verified this and found that SIRT1 protein was downregulated in DR blood samples. Furthermore, we revealed the tendency that SIRT1 was decreased in DR patients’ plasma using the ELISA method (Supplementary Figure 1). Mariani et al. (2015, 2016) and Khalyfa et al. (2019) reported that SIRT1 was downregulated in DM-associated metabolic diseases plasma and the decreased exosome SIRT1 might be correlated with endothelial dysfunction. However, the regulatory mechanism of diabetes to SIRT1 is not clear. Recent studies revealed that the microRNA might be an indispensable regulator (Yamakuchi and Hashiguchi, 2018). Thounaojam et al. (2019) found that overexpression of miR-34a could decrease the expression of SIRT1 directly and induce mitochondrial dysfunction in high glucose-induced retinal endothelial cells. Thus, miR-155-5p, miR-106b, etc. were successively identified to target SIRT1 directly in diabetes conditions (Chen and Yang, 2017; Jiao et al., 2018; Wang et al., 2018). We predicted that SIRT1 was a direct target of miR-29b-3p by the miRNA online database (TargetScanHuman7.2 and miRBase). We also found the different expression patterns of miR-29b-3p/SIRT1 in DR patient and control blood samples. However, the regulatory mechanism of miR-29b-3p to SIRT1 in DR is still unknown. Our *in vitro* research displayed that overexpression of miR-29b-3p in HRMEC downregulated SIRT1 protein expression and promoted cell apoptosis and that the apoptotic promotion of upregulated miR-29b-3p could be rescued by a SIRT1 specific agonist SRT1720. Furthermore, we verified that both miR-29b-3p inhibitor and SRT1720 could increase SIRT1 protein expression and alleviate cell apoptosis in HG-CoCl_2_-induced HRMEC. Therefore, we suggest that SIRT1 is a direct target of miR-29b-3p in DR patients’ retinal microvascular endothelial cells.

In summary, in this study, we investigated the different expression of miR-29b-3p/SIRT1 in blood samples from DR patients and controls. We found that miR-29b-3p was upregulated and SIRT1 was downregulated in DR blood samples. We further explored the interaction mechanism of miR-29b-3p and SIRT1 in cultured HRMEC apoptotic model induced by HG-CoCl_2_. To the best of our knowledge, the relevant reports on the mechanism of miR-29b-3p/SIRT1 in diabetic HRMEC have not yet found. Our study illuminated this mechanism preliminarily. Our data provided the proof that miR-29b-3p/SIRT1 may be a potential therapeutic target for DR. However, DR is a very complicated disease that may be affected by aging, diabetes duration, smoking, etc. (Varma et al., 2014; Chen et al., 2019). We need more clinical samples to verify our findings especially the vitreous or retinal tissues from DR patient surgeries. We need more *in vitro* research on the apoptotic regulation mechanism of miR-29b-3p/SIRT1 via deacetylation method, as epigenetics plays an important role in DM and its complications (Menzies et al., 2016).

## Data Availability Statement

All datasets generated for this study are included in the article/Supplementary Material.

## Ethics Statement

The studies involving human participants were reviewed and approved by the Ethics Committee of Aier eye Hospital. The patients/participants provided their written informed consent to participate in this study.

## Author Contributions

ST and JC conceived the project. YZ carried out most of the experiments and wrote the manuscript. JL assisted in the cell culture and Western blotting. ZC helped in data analysis. All authors approved the final version of the manuscript.

## Conflict of Interest

The authors declare that the research was conducted in the absence of any commercial or financial relationships that could be construed as a potential conflict of interest.
