# Supplementary material for: MicroRNA-29b-3p Promotes Human Retinal Microvascular Endothelial Cell Apoptosis via Blocking SIRT1 in Diabetic Retinopathy
Source: Front Physiol. 2020 Jan 29;10:1621. doi: 10.3389/fphys.2019.01621 (PMC7000655; doi:10.3389/fphys.2019.01621)
Supplement: Supplementary file 1 [file Data_Sheet_1.pdf]

Supplementary Material

Plasma SIRT1 expression using the ELISA method

Method:

Plasma SIRT1 expression was assayed with an ELISA kit (Cusabio). Briefly, we added plasma samples to the detecting wells and incubated for 2 hours at 37°C. After incubating, the liquid was removed and Biotin-antibody was added to each well. After Incubating for 1 hour at 37°C, each well solution was aspirated and washing three times with wash solution. We added HRP-avidin to each well and incubated for 1 hour at 37°C. Aspirating again in each well and washing five times as above procedure. Then 90µl of TMB Substrate was added to each well. After incubating for 20 minutes at 37°C, we added 50µl of Stop Solution to each well. The optical density of each well was acquired by a Synergy™ HTX Multi-Mode Microplate Reader (Bio-Tek Technologies, Winooski, Vermont) within 5 minutes.

Result:

Plasma SIRT1 expression in NC and DR plasma samples. The mean plasma SIRT1 concentration in control group was 0.79ng/ml, while the concentration in DR group was 0.50ng/ml. The expression of SIRT1 in DR group was downregulated, however, the difference had no statistical significance (p=0.1645).

Figure:

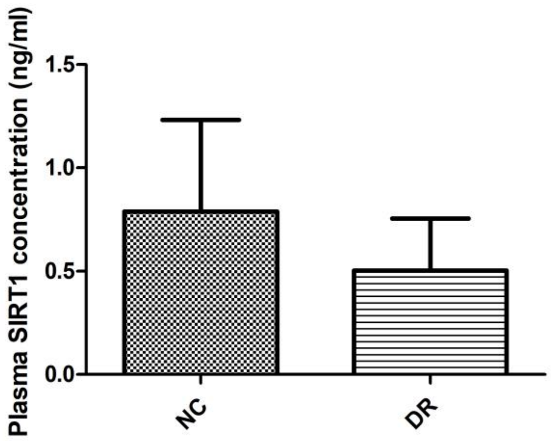

Supplementary Figure 1: Plasma SIRT1 expression using the ELISA method. The mean plasma SIRT1 concentration in control group was 0.79ng/ml, while the concentration in DR group was 0.50ng/ml. The expression of SIRT1 in DR group was downregulated, however, the difference had no statistical significance (p=0.1645). NC, negative control; DR, diabetic retinopathy.
